# Effects of acupuncture on gastrointestinal diseases and its underlying mechanism: a literature review of animal studies

**DOI:** 10.3389/fmed.2023.1167356

**Published:** 2023-06-07

**Authors:** Min-Joon Kim, Seri Lee, Seung-Nam Kim

**Affiliations:** College of Korean Medicine, Dongguk University, Goyang, Republic of Korea

**Keywords:** gastrointestinal diseases, stomach, acupuncture, animal studies, literature review

## Abstract

Acupuncture is a non-pharmacological traditional Chinese medical technique that has been used for various types of gastrointestinal (GI) diseases in Eastern medicine. However, the specific mechanisms underlying acupuncture treatment in the GI tract have not yet been elucidated. In this study, we searched the electronic databases PUBMED, EMBASE, and MEDLINE and identified 30 eligible studies that were summarized in this review. This review demonstrates that treatments, including both manual and electroacupuncture, have therapeutic mechanisms in diverse GI diseases. The underlying mechanisms are broadly divided into the following: changes in gene expression in the gastric mucosa or nuclei of the solitary tract, metabolic change induction, regulation of anti-inflammatory substances, vagal activity increase, change in functional connectivity between brain regions, and control of the number of neurons related to GI diseases. Although this study is limited in that it does not represent all types of GI diseases with different acupuncture methods, this study identified acupuncture as effective for GI diseases through various biological mechanisms. We hope that our study will reveal various mechanisms of acupuncture in GI diseases and play an important role in the therapy and treatment of GI diseases, thus advancing the field of study.

## 1. Introduction

As society evolves rapidly, changes in lifestyle and diet affect the occurrence and development of gastrointestinal (GI) diseases (5). GI disease, a source of morbidity, mortality, and financial burden, has a high frequency and significant socioeconomic impact on the community (4, 3). With the increase in number of diagnostic techniques used, several types of GI disease are identified, which range from mild diseases, such as acute gastritis and acute gastroenteritis, to serious problems, including gastroesophageal reflux disease, inflammatory bowel disease, and upper GI cancer (2). Therefore, medical treatment of GI diseases is important, and the development of therapy is typically required.

Acupuncture, a traditional Chinese medicine, has been used in medical practice (7), and its clinical effects have been reported (8). Therefore, acupuncture has been performed under pathological conditions of GI diseases (1). The effectiveness of acupuncture for gastroparesis has been proven in clinical trials (6). Thus, a recent study has demonstrated the therapeutic effect of acupuncture on GI through the inhibition of gastric acid secretion and GI motility control via different pathways (11). Animal and clinical studies have evaluated the efficacy of acupuncture on GI diseases (10). However, no studies have investigated the mechanisms underlying the therapeutic effects of acupuncture on GI diseases.

This study aimed to comprehensively review the published studies by conducting a comprehensive and systematic search, and to determine the therapeutic effect of acupuncture on GI diseases.

## 2. Methods

### 2.1. Study selection

**Figure 1:**
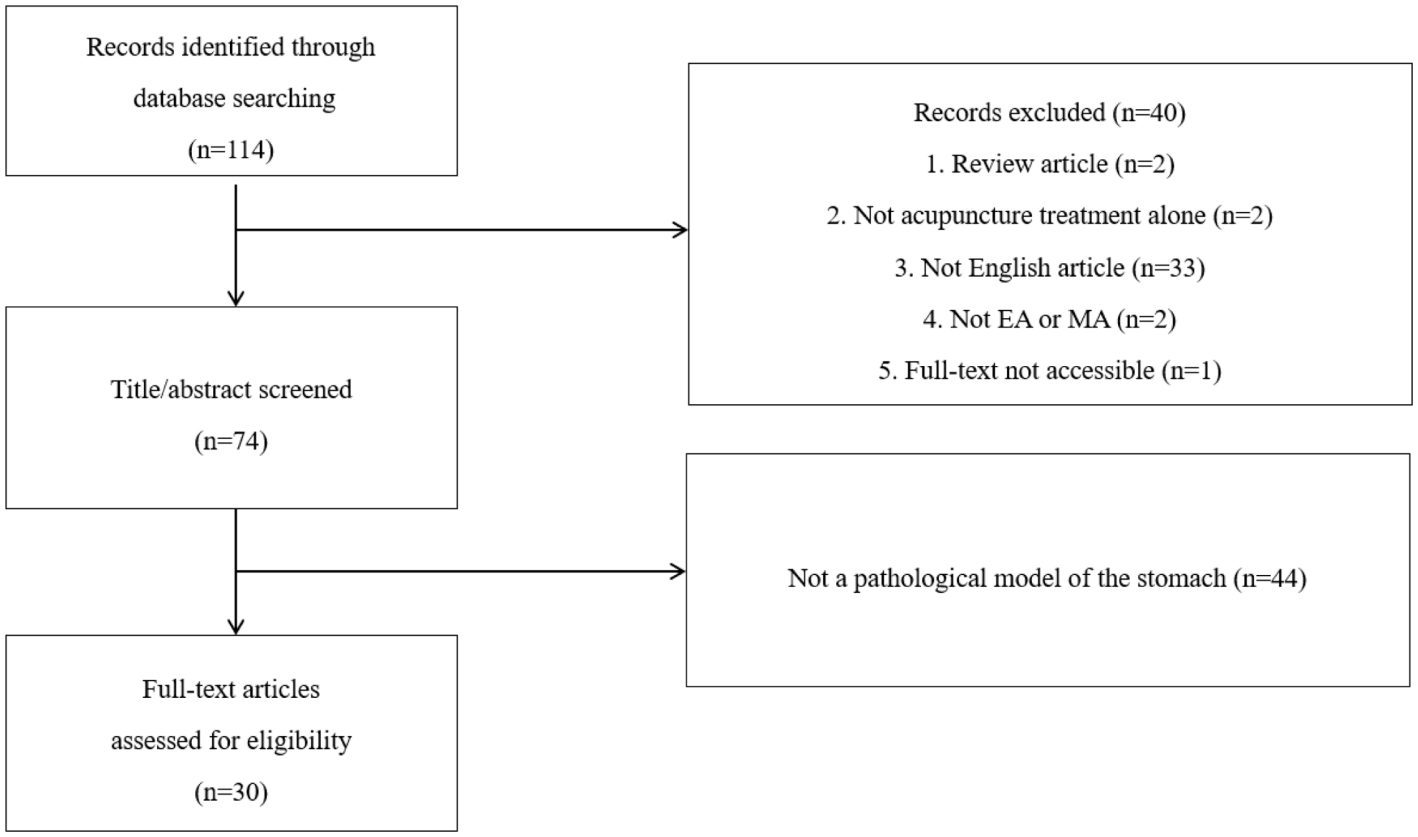
Flowchart of the study.

Figure 1 presents a flowchart of the study selection process for this review. All qualified studies included the keywords acupuncture, animal, and stomach. The databases PUBMED, EMBASE, and MEDLINE were searched using the search terms “(acupuncture [title/abstract] OR electroacupuncture [title/abstract]) AND (animal [title/abstract] OR rat [title/abstract] OR mouse [title/abstract] OR rats[title/abstract] OR mice [title/abstract]) AND (stomach [title/abstract]).” Two reviewers independently conducted the research in this study and identified 114 studies related to the topic. Of the studies, 83 were excluded for the following reasons: review articles, no acupuncture treatment alone, non-English article, no electroacupuncture (EA) or manual acupuncture (MA), full text was not accessible, and study not using a pathological model. Finally, 30 articles were included in the study.

### 2.2. Quality assessment

Quality assessment was performed by two independent researchers using a total of 10 questions, as previously described (9). For the sequence generation domain, the description of whether studies generate allocation sequences in sufficient detail was assessed. The item of baseline characteristics was whether prognostic factors or animal characteristics were described to determine the similarity between the intervention and control groups. For allocation concealment, whether it describes the method used to conceal the allocation sequence in detail was evaluated. Next, the item on random housing describes all measures of housing animals randomly in animal rooms. The fifth item is whether it describes all measures used to blind trial caregivers and researchers from knowing which intervention each animal has received. The item on random outcome assessment asks whether animals were randomly selected for outcome assessment and which methods were used to select the animal. The seventh item is whether it describes all measures used to blind outcome assessors from knowing which intervention each animal has received. Additionally, the item for incomplete outcome data is whether the analysis describes the completeness of the outcome data for each main outcome, including the number or reason for attrition and exclusion. For the selective outcome reporting domain, we assessed whether the examination method of selective result reporting and the identified contents were explained. Finally, for the other sources of the bias domain, we evaluated whether the acupuncture treatment method was specifically explained. Among the 10 questions, the papers scoring 2, 1, and 0 points for each question were considered as having low, unclear, and high risks of bias, respectively.

## 3. Results

### 3.1. Study characteristics and quality assessment

The disease models, animals, acupuncture methods, and acupuncture points used in each study are listed in Table 1. The 30 papers had the following disease models: five gastroparesis models caused by diabetes mellitus, four functional dyspepsia (FD) models, two gastric ulcer models, one peptic ulcer disease (PUD) model, one GI dysmotility model caused by chronic complicated stress, one GI dysmotility model caused by postoperative ileus, two chronic atrophic gastritis (CAG) models, five gastric distention (GD) models, two gastric mucosal injury models, three gastric mucosal lesion (GML) models, two gastric dysrhythmia models, one reduced gastric electrical activity model caused by acute myocardial ischemia, and one acute inflammatory stomach ache model. In total, 26 papers used EA, and 4 papers used MA. The acupuncture points ST2, ST6, ST21, ST36, ST37, CV12, GB14, GB24, GB34, PC5, PC6, SP4, LI10, LI11, SI18, BL21, RN12, and auricular acupunture (ear point ‘stomach’) were used.

**Table 1:** Study characteristics.

GI type	Study ID	Disease model	Animal	Acupuncture method	Acupuncture point
GI dysfunction	Zhou, et al. 2017	FD	mice	Set 1: 100 Hz, 0.3 ms, 0.4–0.5 mA, EA Set 2: 25 Hz, 0.5 ms, 0.4–0.5 mA, EA	Auricular Acupuncture (ear point ‘stomach’)
	Zhang, et al. 2018	FD	rats	25 Hz, 4 mA, EA	ST36
	Zhang, et al. 2020	FD	rats	25 Hz, 4 mA, EA	ST36
	Chen, et al. 2021	FD	rats	2/15 Hz, 1.5 mA, EA	CV12, BL21
	Yang, et al. 2021	CCS	rats	10 Hz, 3 V, EA	ST36
	Murakami, et al. 2019	Postoperative ileus	rats	ST36: 25 Hz, 4 mA, EA and PC6: 100 Hz, 1 mA, EA	ST36, PC6
	Zhang, et al. 1997	Gastric dysrhythmia	rabbits	MA	ST36, PC6
	Song, et al. 2013	Gastric dysrhythmia	rats	25 Hz, 4 mA, EA	ST36
	Wang, et al. 2008	AMI	rats	2/15 Hz, 1–3 mA, EA	PC6, SP4
Gastroparesis	Chen, et al. 2013	Diabetes mellitus	rats	10 Hz, 1–3 mA, LEA and 100 Hz, 1–3 mA, HEA	ST36
	Chen, et al. 2016	Diabetes mellitus	rats	2 Hz, 0.7–1.0 mA, EA	Auricular Acupuncture (ear point ‘stomach’- CO40)
	Tian, et al. 2017	Diabetes mellitus	rats	intermittent: 4 Hz; irregular: 50 Hz, 2-4 V, EA	ST36
	Zhao, et al. 2018	Diabetes mellitus	mice	100 Hz, 1 mA, EA	ST36
	Tian. et al. 2018	Diabetes mellitus	mice	10 Hz, 1–3 mA, LEA and 100 Hz, 1–3 mA, HEA	ST36
Ulcer	Wang, et al. 2005	GU	rats	4 Hz, 0.6 mA, EA	ST36
	Shen, et al. 2019	GU	rats	EA	ST36, ST21
	Li, et al. 2022	PUD	mice	sparse wave: 4 Hz, dense wave: 50 Hz, 2-4 V, 1 mA, EA	ST36, LI10
Gastritis	Liu, et al. 2017	CAG	rats	intermittent: 4 Hz; irregular: 50 Hz, 2 ~ 4 V, EA	ST21, ST36
	Xu, et al. 2017	CAG	rats	100 Hz, 0.4–0.5 mA, EA	ST2, ST21, ST 36
	Xu, et al. 2012	Acute inflammatory stomachache	rats	4–16 Hz, 1 V ~ 5 V, EA	ST36
Gastric distention	Li, et al. 2002	GD	rats	2 Hz, 1–2 mA, EA	PC5, PC6, ST36, ST37
	Liu, et al. 2004	GD	rats	4 and 20 Hz, 1–20 mA, EA	GB14, ST2, ST6
	He, et al. 2006	GD	rats	MA	ST2
	Hong, et al. 2015	Acute GD	rats	2,3 Hz MA	ST36
	Liang, et al. 2018	GD	rats	MA	ST36, LI11, BL21
Injury	Li, et al. 2006a	Gastric mucosal injury	rats	intermittent: 4 Hz, irregular: 20 Hz, 6-15 V, EA	ST2, ST21, ST36, GB14, GB24, GB34
	Yang, et al. 2006	Gastric mucosal injury	rats	intermittent: 4 Hz; irregular: 20 Hz, EA	ST2, ST21, ST36, GB14, GB24, GB34
	Li, et al. 2006b	GML	rats	intermittent: 4 Hz; irregular: 50 Hz, 3 V, EA	ST2, ST21, ST36, GB14, GB24, GB34
	Xu, et al. 2015	GML	rats	intermittent: 4 Hz; irregular: 50 Hz, 2-4 V, EA	ST2, ST21, ST36, GB14, GB24, GB34
	Huang, et al. 2022	GML	rats	intermittent: 4 Hz, irregular: 50 Hz, EA	ST36, ST21

FD, functional dyspepsia; GU, gastric ulcer; PUD, peptic ulcer disease; CCS, chronic complicated stress; CAG, chronic atrophic gastritis; GD, gastric distention; GML, gastric mucosal lesion; AMI, acute myocardial ischemia; MA, manual acupuncture; EA, electroacupuncture; LEA, low-frequency EA; HEA, high-frequency EA.

**Figure 2:**
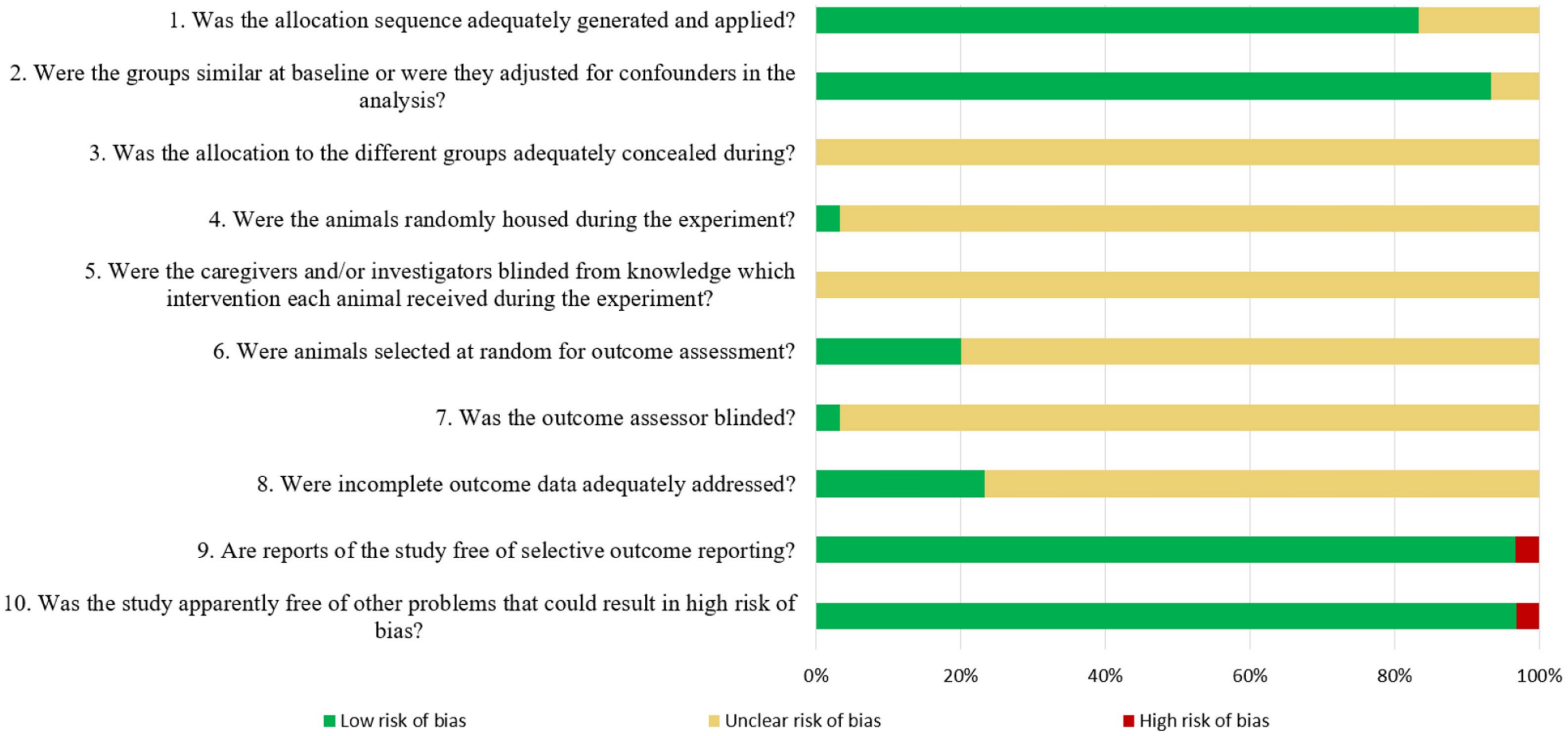
Quality assessment of the literatures.

Figure 2 presents the results of the quality assessment of the 30 research papers. Two papers received 12 points, four papers received 13 points, 16 papers received 14 points, six papers received 15 points, one paper received 16 points, and one paper received 17 points.

### 3.2. Main text

Table 2 summarizes the efficacy and mechanism of acupuncture treatment for GI diseases.

**Table 2:** Therapeutic effects and underlying mechanisms of the studies.

GI type	Study ID	Therapeutic effect	Mechanism
GI dysfunction	Zhou, et al. 2017	treat FD only set 1 (100 Hz, 0.3 ms, 0.4–0.5 mA, EA): gastric hypersensitivity ↓	vagal activity ↑, sympathovagal imbalance ↓
	Zhang, et al. 2018	FD ↓, gastric emptying ↑	plasma NE ↓, vagal activity ↑, sympathetic activity ↓, ACh ↑
	Zhang, et al. 2020	GSW ↑, FD ↓	plasma NE ↓, ACh ↑, c-fos expression in the NTS ↑, vagal activity ↑, sympathovagal ratio ↓
	Chen, et al. 2021	FD ↓, gastric emptying ↑	FC between insula and brain regions ↑ (S1, hippocampal CA3, PoDG, CPu, and PnO), FC between insula and brain regions ↓ (bilateral M1/2, PVA, and LC)
	Yang, et al. 2021	GI dysmotility ↓, gastric emptying ↑	central NPY expression ↑, CRF expression ↓, plasma corticosterone level ↓, HPA axis activities ↓
	Murakami, et al. 2019	GI dysmotility ↓, gastric emptying ↑	plasma TNF-α ↓, sympathovagal balance ↓, vagal activity ↑
	Zhang, et al. 1997	treat gastric dysrhythmia, percentage of disturbance gastric electric slow wave ↓, frequency of gastric electric slow wave ↑	N/A
	Song, et al. 2013	gastric dysrhythmia ↓, gastric emptying ↑, GSW ↑	plasma IL-6 ↓, vagal activity ↑
	Wang, et al. 2008	recovery of gastric electrical activities, amplitude and frequency of slow waves of EGG ↑	NOS expression in antrum ↑
Gastroparesis	Chen, et al. 2013	gastroparesis ↓, gastric emptying ↑	c-kit in gastric wall↑, c-kit positive ICC-IM, ICC-MY and ICC-SM ↑
	Chen, et al. 2016	gastroparesis ↓, GI motility ↑	nNOS in antrum ↑
	Tian, et al. 2017	gastroparesis ↓, gastric emptying ↑	c-kit in the antrum and corpus ↑, mSCF in the antrum and corpus ↑, ETV1 in the antrum and corpus↑
	Zhao, et al. 2018	gastroparesis ↓, gastric emptying ↑	SDF-1 in the stomach and serum ↑, CXCR4 in the stomach ↑, c-kit in the stomach ↑, mSCF in the stomach↑, pERK in the stomach ↑, ETV1 in the stomach ↑, SDF-1/CXCR4 and mSCF/kit-ETV1 signaling pathways
	Tian. et al. 2018	gastroparesis ↓, gastric emptying ↑	ICC density ↑, HO-1 positive M2 macrophages in the stomach ↑, M1 macrophages in the stomach ↓, IL-10 in the stomach ↑, serum MDA ↓
Ulcer	Wang, et al. 2005	GU ↓	c-fos ↓
	Shen, et al. 2019	GU ↓	isoleucine ↑, valine ↑, glycerol ↑, serine ↑, metabolic changes such as neurotransmitter metabolism, cell metabolism, energy metabolism, antioxidation, and tissue repair
	Li, et al. 2022	PUD ↓, restore the structure of gastric microbiota	serum DA↑ and TFF↑
Gastritis	Liu, et al. 2017	CAG ↓	substance P ↑, ghrelin ↑, methionine ↑, betaine ↑, phenylalanine ↑, ethanolamine ↑, inositol ↑, glycogen ↓, glucose ↓, glutathione ↓, hypoxanthine ↓, glutamine↓, methylmalonate ↓, malonic acid ↓, inosine ↓, nicotinamide ↓
	Xu, et al. 2017	malignant proliferation of gastric mucosal cells ↓, CAG ↓	PCNA ↓, Ag-NORs ↓, EGF ↓, VEGF ↓, c-myc and NF-κB genes expression ↓, upregulation and downregulation of 8 (GPC, betaine, lactate, PC, taurine, ethanolamine, glutamate, and formate) and 2 metabolites (myo-inositol and β-glucose), cell proliferation factor ↓
	Xu, et al. 2012	stomachache ↓, paroxysmal contraction wave ↓, sustain low-amplitude activity	NOS-positive neurons ↑, AChE-positive perikarya and enzymatic activity ↑, VIP-positive nerve fibers ↑, CGRP-positive crude fibers ↓
Gastric distention	Li, et al. 2002	GD ↓	pressor reflex ↓
	Liu, et al. 2004	gastrointestinal pain ↓, GD ↓	c-fos ↑
	He, et al. 2006	GD ↓	activate 33 GD-related neurons
	Hong, et al. 2015	GD ↓	spike frequency of excitatory gastric-related WDR neurons in SDH ↓
	Liang, et al. 2018	GD ↓	alter the discharge of GD-responsive MVN neurons
Injury	Li, et al. 2006a	GUI ↓	gastric mucosal intestinal TFF ↑
	Yang, et al. 2006	gastric mucosal injury ↓	EGFR in gastric mucosal cells ↑
	Li, et al. 2006b	GUI ↓	gastric mucosal intestinal TFF ↑
	Xu, et al. 2015	GML, GU ↓ (ulcer scores of gastric mucosa ↓)	acetate ↑, 3-HB ↑, hippurate ↑, creatine ↑, phosphocholine ↑, DMG ↑, PAG ↑, NAG ↓, ACh ↓, α-KG ↓, MN ↓, trigonelline ↓
	Huang, et al. 2022	GML ↓, gastric mucosal inflammation ↓	NF-κB p65 in gastric mucosa ↓, CD3 in gastric mucosa ↓, changes metabolites in medulla and in the cerebral cortex

FD, functional dyspepsia; GU, gastric ulcer; PUD, peptic ulcer disease; CCS, chronic complicated stress; CAG, chronic atrophic gastritis; GD, gastric distention; GML, gastric mucosal lesion; AMI, acute myocardial ischemia; GI, gastrointestinal; GSW, gastric slow wave; GUI, gastric mucosal lesion index; EGG, electrogastrogram; NOS, nitric oxide synthase; MDA, malondialdehyde; NE, norepinephrine; Ach, acetylcholine; FC, functional connectivity; S1, primary somatosensory cortex; PoDG, polymorphic layer of dentate gyrus; CPu, caudate putamen; PnO, oral pontine reticular nuclei; M1/2, primary and secondary motor cortexes; PVA, paraventricular hypothalamic nucleus; LC, limbic cortex; DA, dopamine; TFF, trefoil factor; NPY, neuropeptide Y; CRF, corticotropin-releasing factor; HPA, hypothalamus-pituitary–adrenal; PCNA, proliferating cell nuclear antigen; Ag-NORs, Ag-nucleolar organization regions; EGF, epidermal growth factor; VEGF, vascular endothelial growth factor; PC, phosphocholine; WDR, wide dynamic range; SDH, spinal dorsal horn; MVN, medial vestibular nucleus neurons; EGFR, epidermal growth factor receptor; 3-HB, 3-hydroxybutyrate; DMG, N,N-dimethylglycine; PAG, phenylacetylglycine; NAG, N-acetylglutamate; α-KG, α-ketoglutarate; MN, 1-methylnicotinamide; VIP, vasoactive intestinal peptide; CGRP, calcitonin gene-related peptide.

#### 3.2.1. GI dysfunction

FD is a chronic dysfunction of the GI tract that is difficult to treat and affects social function and quality of life (17). Its main symptoms include epigastric pain, epigastric burns, postprandial fullness, and initial satiety without any specific cause (12). EA upregulates the vagal nerve and downregulates sympathetic activity, thereby reducing gastric hypersensitivity (13). Moreover, EA lowers plasma norepinephrine (NE) and acetylcholine (ACh), resulting in the activation of vagal activity and lower sympathetic activity (15, 16). EA also increases c-Fos expression in the nuclei of the solitary tract (NTS) and increases the gastric slow wave (GSW) (16). In another study, EA changed the functional connectivity (FC) between the insula and brain regions. The regions with increased FC are the primary somatosensory cortex, hippocampal CA3, polymorphic layer of the dentate gyrus, caudate putamen, and oral pontine reticular nuclei, and the regions with decreased FC are the bilateral primary and secondary motor cortices, paraventricular hypothalamic nucleus, and limbic cortex (14).

GI dysmotility is characterized by delayed gastric emptying, which frequently occurs in critically ill patients (18). Patients with GI dysmotility exhibit various non-discriminatory symptoms including vomiting, abdominal pain, abdominal distention, and constipation (21). In chronic complicated stress models, EA increased the expression of central neuropeptide Y (NPY) and gamma-aminobutyric acid (GABA) A receptor and decreased central corticotropin-releasing factor (CRF), plasma corticosterone levels, and hypothalamus–pituitary–adrenal (HPA) axis activity. NPY inhibits CRF, which inhibits gastric motility through GABA receptors, reduces HPA axis activity, and further reduces plasma corticosterone levels to restore gastric motility (22).

Postoperative ileus is a widely known complication characterized by transient impairment of GI function after abdominal surgery (19). In a postoperative ileus model, EA increased gastric emptying and treated GI dysmotility by decreasing the sympathetic balance and increasing vagus nerve activity. EA also increases the release of ACh, which inhibits the release of TNF-α, an inflammatory factor, through the α7 nicotinic acetylcholine receptor (20).

Gastric dysrhythmia represents the underlying cause of nausea; thus, it is an objective biomarker of nausea (26). In a gastric dysrhythmia model, MA decreased the percentage of disturbance of gastric electric slow waves, increased the frequency of gastric electric slow waves, and treated gastric dysrhythmia (23). Additionally, EA lowered plasma IL-6 levels, activated vagal activity, and increased gastric emptying and GSW in a gastric dysrhythmia model (25).

The electrical activity of the stomach serves as the basis for GI motility, which functions in digestion, absorption, excretion, and protection (27). In an acute myocardial ischemia model, EA restored gastric electrical activity, increased nitric oxide synthase (NOS) expression in the stomach, and increased the amplitude and frequency of slow waves in the electrogastrogram. The generated NO activates guanylate cyclase, and the resulting cyclic guanosine monophosphate can exert biological effects such as increasing gastric electrical activity by regulating myocardial blood pumping (24).

#### 3.2.2. Gastroparesis

Gastroparesis is characterized by delayed gastric emptying and upper GI symptoms (28). Its symptoms include nausea, vomiting, early satiety, postprandial fullness, bloating, and upper abdominal pain (29). All studies on gastroparesis used a diabetes mellitus-induced gastroparesis model (31–33). Interestingly, the c-Kit signaling pathway is a common therapeutic mechanism in acupuncture treatment for gastroparesis. EA also expressed the tyrosine kinase receptor c-Kit in the gastric wall and restored the ICC network, leading to decreased inflammation (31). Moreover, EA increases the expression of nNOS in the antrum, thus increasing GI motility (30). In another diabetes mellitus model, EA increased the c-Kit, mSCF, and ETV1 expressions in the antrum and corpus, thereby accelerating gastric emptying (34). Moreover, EA increased the SDF-1, CXCR4, and pERK expressions in stomach tissues (32). EA increased the density of ICC and the expression of HO-1 positive M2 macrophages and decreased that of M1 macrophages. Additionally, EA increases the IL-10 expression and decreases serum malondialdehyde levels (33).

#### 3.2.3. Ulcer

Gastic ulcer (GU) is defined as any damage to the mucosa of the stomach lining, which extends >5 mm in diameter and pierces through the muscular mucosa (39). Studies on GU have demonstrated that acupuncture induces metabolic changes and increases vagal activity. EA decreased the expression of c-Fos, which serves as a marker for activity in the central nervous system and can be induced by GU. The vagus nerve mediates c-Fos generation in the NTS, parasympathetic afferents promote harmful visceral stimulation, and EA prevents the formation and increase of Fos-Li neuronal products (38). In the other GU model, isoleucine, valine, glycerol, and serine levels increased regardless of the EA treatment time. EA has therapeutic effects on GU by causing several metabolic changes such as neurotransmitter metabolism, cell metabolism, energy metabolism, antioxidation, and tissue repair (37).

PUD is an interruption of the lining of the GI tract lining due to abnormal secretion of gastric acid or pepsin (36). EA restores the structure of the gastric mucosa and muscle layer by increasing the levels of dopamine and trefoil factor (TFF). EA also improves the diversity of gastric microbiota, such as *Firmicutes* and *Bacteroidetes*, through which EA exerts therapeutic effects on PUD (35).

#### 3.2.4. Gastritis

CAG is the result of an inflammatory process that ultimately leads to the loss of appropriate mucosal glands (41). In the CAG model, EA repairs the nervous system of the stomach and brain, which may modulate energy metabolism, membrane metabolism, and various neurotransmitters in the nervous system (40). EA also blocked the malignant proliferation of gastric mucosal cells by reducing the expression of epidermal growth factor, vascular endothelial growth factor, proliferating cell nuclear antigen, Ag-nucleolar organization regions, c-Myc, and NF-κB in gastric mucosal cells. Additionally, eight metabolites (glycerophosphocholine, lactate, phosphocholine, myo-inositol, ethanolamine, glutamate, formate, and β-glucose) were increased, and two metabolites (betaine and taurine) were decreased in gastric tissue by EA (42).

Corrosive substances cause severe GI inflammation and acute stomach pain (43). In a model of acute gastritis caused by corrosive substances, EA controlled GI motility by increasing the number of NOS-positive neurons in the GI wall and restoring ACh activity. EA also increased the number of vascular active intestinal peptide-positive nerve fibers and decreased the number of calcitonin gene-related peptide-positive crude fibers, which affect the smooth muscle relaxation function of the GI (44).

#### 3.2.5. Gastric distention

Normally, the gastric mechanical expansion signal occurs after meals and induces GD (45). However, in pathological situations, abnormal GD can be caused by air entering the stomach during strong or fast rescue breathing (49). EA reduces the pressor reflex and has a therapeutic effect on GD. This study suggests that EA inhibits GD-induced pressor reflex, which includes interactions between afferent inputs in various regions of the brain (50). In another GD model, EA reduced GI pain by increasing the c-Fos expression in the NTS and demonstrated therapeutic effects in GD (57). Interestingly, acupuncture also regulates the number of neurons associated with GD (47–55). MA exhibited a therapeutic effect on GD by activating 33 of 47 GD-relative neurons (47) and by lowering the spike frequency of excitatory gastric-related wide dynamic range neurons in the spinal dorsal horn, which is increased by acute GD (53). Moreover, MA alters the discharge of GD-responsive medial vestibular nucleus neurons (55).

#### 3.2.6. Injury

Gastric mucosal injury or GML is caused by an imbalance in a series of defense mechanisms that protect the mucous membrane from external aggressive factors (51). EA restores gastric mucosal injury by increasing the expression of epidermal growth factor receptors in gastric mucosal cells (48). In another gastric mucosal injury model, EA decreased the GML index and increased intestinal TFF expression in the gastric mucosa, which restored the wound to the GI mucosa (46, 52). In the GML model, the ulcer scores of the gastric mucosa were decreased by EA. EA increases acetate, 3-hydroxybutyrate, hippurate, creatine, phosphocholine, N,N-dimethylglycine, phenylacetylglycine, N-acetylglutamate, and Ach levels. Additionally, EA lowers α-ketoglutarate, 1-methylnicotinamide, and trigonelline levels (56). In another GML model, EA inhibited gastric mucosal inflammation by lowering the NF-κB p65 and CD3 expressions in the gastric mucosa. Furthermore, 13 metabolite concentrations in the medulla and 9 metabolite concentrations in the cerebral cortex changed after EA (54).

## 4. Discussion

Acupuncture, a complementary and alternative therapy (58), has been used to treat various diseases (59). Its use for GI conditions has been demonstrated in previous studies (60). Clinical trials on acupuncture management for GI diseases have been established (61). However, previous studies are insufficient to clarify the acute therapeutic mechanisms of acupuncture in GI diseases. Therefore, in this study, we aimed to determine the underlying mechanism of acupuncture treatment for GI diseases in animal models.

In our report, 30 studies were selected, and we divided the various types of GI diseases of the included studies into six categories of disease groups (GI dysfunction, gastroparesis, ulcers, gastritis, GD, and injury). Moreover, the therapeutic mechanisms of acupuncture that were obtained from our investigation were changes in gene expression, regulation of anti-inflammatory substances, metabolic changes, increase in vagal activity, change in FC between the insula and brain regions, and control of neuron numbers. Interestingly, studies included in the gastroparesis group identified a common mechanism of the c-Kit signaling pathway. Meanwhile, some mechanisms, such as downregulation of plasma NE and upregulation of ETV1 expression, were duplicated in different studies. Although the present review has a deficiency in representing the entire therapeutic mechanism of acupuncture treatment for GI diseases, this study is meaningful because it provides an overview of the underlying mechanism of acupuncture therapy on stomach-related problems and gastric disease for the first time.

Among the 30 studies, 26 used EA and 4 used MA. The methods of acupuncture treatment differs between MA and EA. EA methods include frequency, waveform, time, and current intensity, whereas MA methods include twisting, twirling, thrusting, lifting, or a combination of these motions (61). Therefore, the efficacy of acupuncture also changes depending on the method used (62). In our study, one study has reported the opposite outcome of the mechanism of different acupuncture methods depending on the frequency of acupuncture stimulation. The spike frequency was further excited by 0.5 Hz and 1 Hz MA stimulation. Meanwhile, the activity significantly decreased for 2 Hz and 3 Hz MA stimulation (53). By contrast, another study has revealed an equivalent mechanism, although the acupoints and acupuncture stimulation were different (20). According to our study results, biological mechanisms exist or change under different conditions of disease and acupuncture methods. Further studies are needed to clarify the therapeutic mechanism of acupuncture in GI diseases and to reveal the difference in the mechanism depending on the acupuncture method.

This study aimed to investigate the effect of acupuncture treatment on stomach-related problems and to clarify the therapeutic mechanism. Although our study is limited by the study size and diverse types of GI diseases, our findings are significant because various mechanisms of the effects of acupuncture on GI diseases have been demonstrated. Further research on the role of acupuncture in GI diseases with a larger sample size is necessary. Therefore, our study results will help reveal the potential mechanisms and therapeutic effects of acupuncture for GI diseases in the future. We expect that our report will contribute to further experimental and clinical trials of acupuncture treatment for stomach-related diseases and digestive system disorders.

## Author contributions

M-JK and SL wrote the paper. M-JK investigated the study. S-NK designed and supervised the study. SL and S-NK analyzed the data and revised the paper. All authors contributed to the article and approved the submitted version.

## Funding

This work was supported by the National Research Foundation of Korea funded by the Korean government (MSIT; NRF-2020R1C1C1004107) and from the Ministry of Health and Welfare through the Korea Health Industry Development Institute (KHIDI; grant no. HF21C0018).

## Conflict of interest

The authors declare that the research was conducted in the absence of any commercial or financial relationships that could be construed as a potential conflict of interest.

## Publisher’s note

All claims expressed in this article are solely those of the authors and do not necessarily represent those of their affiliated organizations, or those of the publisher, the editors and the reviewers. Any product that may be evaluated in this article, or claim that may be made by its manufacturer, is not guaranteed or endorsed by the publisher.
